# Sustainable solutions to the continuous threat of antimicrobial resistance

**DOI:** 10.1093/haschl/qxaf012

**Published:** 2025-01-24

**Authors:** Brad Spellberg, David N Gilbert, Michael Baym, Gonzalo Bearman, Tom Boyles, Arturo Casadevall, Graeme N Forrest, Sarah Freling, Bassam Ghanem, Fergus Hamilton, Brian Luna, Jessica Moore, Daniel M Musher, Travis B Nielsen, Priya Nori, Matthew C Phillips, Liise-Anne Pirofski, Andrew F Shorr, Steven Y C Tong, Todd C Lee, Emily G McDonald

**Affiliations:** Hospital Administration, Los Angeles General Medical Center, Los Angeles, CA 90033, United States; Division of Infectious Diseases, Department of Medicine, Oregon Health Sciences University School of Medicine, Portland, OR 97239, United States; Departments of Biomedical Informatics and Microbiology, Harvard School of Medicine, Boston, MA 02115, United States; Department of Medicine, Division of Infectious Diseases, Virginia Commonwealth University, Richmond, VA 23298, United States; Clinical HIV Research Unit, University of the Witwatersrand, Johannesburg 2017, South Africa; Department of Microbiology and Immunology, Johns Hopkins University School of Medicine, Baltimore, MD 21205, United States; Division of Infectious Diseases, Rush University Medical Center, Chicago, IL 60612, United States; Division of Infectious Diseases and Epidemiology, Department of Medicine, Los Angeles General Medical Center, Los Angeles, CA 90033, United States; Pharmaceutical Care Department, King Abdulaziz Medical City, National Guard Health Affairs, Jeddah 14611, Saudi Arabia; MRC Integrative Epidemiology Unit, University of Bristol, Bristol BS8 1QU, United Kingdom; Infection Science, North Bristol NHS Trust, Bristol BS8 1QU, United Kingdom; Department of Molecular Microbiology and Immunology, Keck School of Medicine at USC, Los Angeles, CA 90033, United States; Department of Pharmacy, Desert Regional Medical Center, Palm Springs, CA 92262, United States; Departments of Medicine and Molecular Virology, Immunology Baylor College of Medicine, Houston, TX 77030, United States; Medical Care Line, Michael E. DeBakey VA Medical Center, Houston, TX 77030, United States; Department of Molecular Microbiology and Immunology, Keck School of Medicine at USC, Los Angeles, CA 90033, United States; Division of Infectious Diseases, Department of Medicine, University of California San Diego, San Diego, CA 92093, United States; Department of Medicine, Division of Infectious Diseases, Albert Einstein College of Medicine, Bronx, NY 10461, United States; Departments of Biomedical Informatics and Microbiology, Harvard School of Medicine, Boston, MA 02115, United States; Division of Infectious Diseases, Department of Medicine, Massachusetts General Hospital, Boston, MA 02114, United States; Department of Medicine, Division of Infectious Diseases, Albert Einstein College of Medicine, Bronx, NY 10461, United States; Pulmonary and Critical Care Medicine, Medstar Washington Hospital Center, Washington, 20010 DC; Victorian Infectious Diseases Service, The Royal Melbourne Hospital, at the Peter Doherty Institute for Infection and Immunity, Melbourne 3052, Australia; Department of Infectious Diseases, The University of Melbourne at the Peter Doherty Institute for Infection and Immunity, Melbourne 3052, Australia; Division of Infectious Diseases, Department of Medicine, McGill University, Montreal, Quebec H4A 3J1, Canada; Division of General Internal Medicine, Department of Medicine, McGill University Health Centre, Montreal, Quebec, Canada

**Keywords:** antimicrobial resistance, antibiotics, US government, Congress, legislation

## Abstract

To combat antimicrobial resistance (AMR), advocates have called for passage of the Pioneering Antimicrobial Subscriptions To End Upsurging Resistance (PASTEUR) Act in the United States, which would appropriate $6 billion in new taxpayer-funded subsidies for antibiotic development. However, the number of antibiotics in clinical development, and US Food and Drug Administration approvals of new antibiotics, have already markedly increased in the last 15 years. Thus, instead of focusing on more economic subsidies, we recommend reducing selective pressure driving AMR by (1) establishing pay-for-performance mechanisms that disincentivize overprescribing of antibiotics, (2) focusing existing research and development funding on strategies that decrease reliance on antibiotics, and (3) changing regulation or law to require specialized training in antibiotic stewardship for a clinician to be able to prescribe new antibiotics that target unmet AMR need. To stabilize the antibiotic market, we recommend (1) establishment of an advisory board of clinical practitioners to more accurately target existing antibiotic incentives and (2) endowment of nonprofit companies that sustainably self-fund antibiotic discovery, creating a bench of molecules that can be partnered with industry at later stages of development.

## Introduction

A recent study predicted that a shocking 92 million deaths associated with antimicrobial resistance (AMR) will occur worldwide between 2025 and 2050.^1^ Advocates cite this number, combined with concerns about an antibiotic market failure, to lobby for the passage of the Pioneering Antimicrobial Subscriptions To End Upsurging Resistance (PASTEUR) Act.^1-3^ The PASTEUR Act has been introduced in 3 consecutive US Congresses (116th–118th). It would appropriate $6 billion in new antibiotic development subsidies in the form of subscription contracts. These subscription contracts would pay pharmaceutical companies a fixed amount of money for each antibiotic approved, no matter how often they are prescribed.

As professionals who care for patients and conduct basic and clinical research on AMR infections, we are concerned about an echo-chamber effect, in which advocates amplify an apocalyptic narrative of AMR-associated deaths due to lack of antibiotics to call for ever increasing taxpayer subsidies.

Here we lay out a longer-term view of the challenges posed by AMR and deaths from infection, and describe solutions that are feasible and economically sustainable.

## Implications of the history of AMR

Antimicrobial molecules, and resistance mechanisms to defeat them, were invented by microbes more than 2 billion years ago (Table 1).^4,5^ During billions of years of evolution, microbes have likely attacked every biochemical pathway that can be targeted by antimicrobials, and created resistance mechanisms to protect all of those pathways.^5^ Thus, no matter what antimicrobial compound humans discover in the future, a resistance mechanism to protect against it likely already exists in nature, or will readily evolve under selective pressure.^5,9,10^

**Table 1.** A brief history of antimicrobial substances.

Years in the past (future)	Event
2 200 000 000	Microbes invent antimicrobial substances
2 200 000 000	Microbes invent AMR^4^
1 000 000 000	Eukaryotes evolve
225 000 000	Mammals appear
300 000	*Homo sapiens* appear
10 000	Civilization appears
92	First safe/effective systemic antimicrobial used by humans (Sulfa, 1932)^6^
80	First “boom” period of antibiotic R&D—1940s–1950s
64	First antibiotic pipeline concern—1960s^7^
54	Second “boom” period of antibiotic R&D—1970s–1990s
24	Second antibiotic pipeline concern—2000s^8^
14	Third “boom” period of antibiotic R&D—2010s–2020s
(??)	Third antibiotic pipeline concern—???

Abbreviations: AMR, antimicrobial resistance; R&D, research and development.

We will never “win a war” against microbes. No “gorilla-cillin” will ever come along to save us from the emergence of AMR. Resistance is inevitable.

Antimicrobial resistance is therefore a continuous threat that requires sustainable solutions. Yet, far from being sustained, the brief history of human discovery of new antibiotics has suffered from repeated boom-and-bust cycles (Table 1). In 1965, after the first discovery boom from 1945–1960, a roundtable of some of the world's experts on AMR was already expressing concern about the collapse of the antibiotic pipeline.^7^ So many similar antibiotics had been developed that the market became saturated, yet AMR kept emerging. These experts presciently wrote, “The needs are multi-fold—to overcome the problems of resistance, to use against gram-negative bacillary infections…more active and less toxic ones for fungal infections, better ones against mycobacteria…and for the prevention and treatment of viral infections.”^7^ That 60-year old quote would be no less accurate had it been published this morning.

Newly emerging AMR led to a second discovery boom from the 1970s–1990s, resulting in the introduction of many modern, cornerstone antibiotics. Once again, this antibiotic boom saturated the market and led to the second pipeline bust in the late 1990s.^8,11^

Thus, history teaches us a critically important lesson: bringing many similar antibiotics to market perpetuates boom-and-bust cycles. As multiple new antibiotics with overlapping spectra of activity compete with each other for market share, diminishing returns on investment occur, suppressing further corporate investment in the antimicrobial space.^11-13^ Emerging AMR does temporarily increase unmet need; however, each resistance mechanism creates only a relatively small market for needed new drugs, and the availability of 1 or 2 new antibiotics is typically adequate to meet that need.

As a solution to low return on investment for antibiotics, the PASTEUR Act seeks to delink industry revenue from new antibiotic sales. Unfortunately, this solution would be unsustainable. Assuming several new antibiotics came to market as a result, resistance to them would develop within a few years, putting society right back in the position of needing billions of dollars of additional subsidies.

What we need are sustainable solutions that avoid perpetuating boom-and-bust cycles, and result in new antibiotics with minimal overlap in spectrum of activity. As the roundtable noted in 1965, “There is a possibility that not many new antibiotics remain to be discovered, and if so it is better that they should be introduced one by one at fairly long intervals.”^7^

## Misconceptions about the current state

### Is the antibiotic pipeline collapsing?

**Figure 1.**
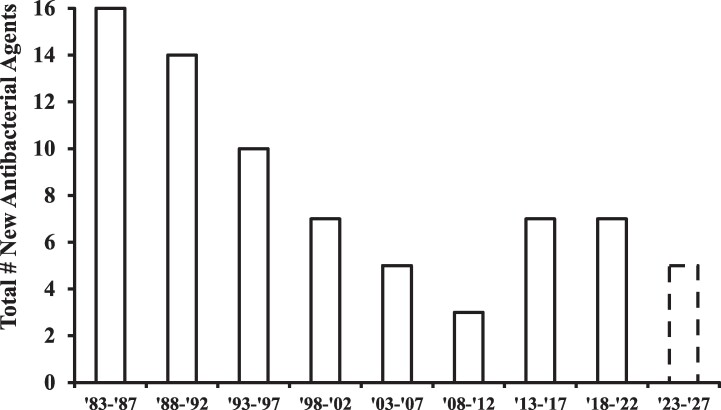
New antibiotics approved by the US Food and Drug Administration (FDA) per 5-year period. The graph groups approvals per 5-year periods starting in 1983 because the dates of new approvals are confirmed from the FDA Orange Book database (https://www.accessdata.fda.gov/scripts/cder/ob/index.cfm), which lists specific dates of approvals from 1983 on. Only systemically active antibacterial agents are included, excluding topical agents, antifungals, antivirals, and anti-parasitics. The final period is indicated with a dashed line because 3 years remain at the time of publication.

In 2004, an exhaustive survey of the drug pipelines in pharmaceutical and biotechnology companies found only 6 new antibiotics in development, which was sadly fewer than the 8 drugs in development to treat bladder hyperactivity, and barely more than the 4 to treat erectile dysfunction.^8^ Since the pipeline is a leading-edge indicator, US Food and Drug Administration (FDA) approvals of new antibiotics fell from 16 between 1983 and 1987 to a paltry 3 between 2008 and 2012 (Figure 1).

Simultaneous to this second historical pipeline collapse, the world experienced the emergence of community-onset methicillin-resistant *Staphylococcus aureus* (MRSA), and a dramatic rise in multidrug-resistant strains of gram-negative bacteria and *Mycobacterium tuberculosis*.^14^ Thus, extensive efforts were made to raise public awareness of AMR in the 2000s.^5,8,11,14-16^ These efforts led to greater recognition of the unmet need in industry, passage of regulatory reform, and establishment of economic “push” incentives to offset the cost and risk of research and development (R&D) for new antibiotics.^5,11,15-17^

The result has been a remarkable resurgence of the antibiotic pipeline. According to the Pew Charitable Trusts, there were 43 antibiotics in clinical development in 2021^18^—an increase of more than 600% since 2004. FDA approvals of new antibiotics have also markedly increased, despite the pause caused by the COVID-19 pandemic (Figure 1 and Table 2). Indeed, in 2024 alone, 4 antibiotics were approved, which is more than during the entire nadir from 2008 through 2012.

**Table 2.** New antibiotics approved by the US FDA since 2000.

Year approved	Drug	Primary unmet need?	Comment
2000	Linezolid	Yes	First new agent to treat MRSA and VRE
2001	Cefditoren	No	Off the market now
2001	Ertapenem	No	Multiple carbapenems on the market
2003	Gemifloxacin	No	Multiple fluoroquinolones on the market; second MRSA agent
2003	Daptomycin	+/-	Third new agent to treat MRSA and second for VRE
2005	Tigecycline	Yes	First new agent to treat CRAB and CRE, fourth MRSA agent
2007	Doripenem	No	Multiple carbapenems on the market
2009	Telavancin	No	Fifth MRSA, third VRE agent
2010	Ceftaroline	No	Sixth MRSA agent
2012	Bedaquiline	Yes	First new drug approved to treat TB in 50 years
2014	Tedizolid	No	Seventh MRSA, fourth VRE agent
2014	Dalbavancin	No	Eighth MRSA, fifth VRE agent
2014	Oritavancin	No	Ninth MRSA, sixth VRE agent
2014	Ceftolozane-tazobactam	Yes	First new drug for CRPA
2015	Ceftazidime-avibactam	Yes	Second new drug for CRE
2017	Delafloxacin	No	Tenth MRSA agent, multiple fluoroquinolones on the market
2017	Meropenem-vaborbactam	No	Third new CRE drug
2018	Plazomicin	No	Fourth new CRE drug; eleventh MRSA agent
2018	Eravacycline	No	Fifth new CRE drug, second new CRAB drug
2018	Omadacycline	+/–	Twelfth MRSA agent; may be useful for resistant NTM, but no high-quality studies to validate this
2019	Imipenem-relebactam	No	Sixth new CRE drug
2019	Pretomanid	Yes	New TB drug, developed by nonprofit (TB Alliance)
2019	Lefamulin	No	Thirteenth MRSA agent, numerous pneumonia drugs on the market
2019	Cefiderocol	Yes	Seventh new CRE drug but first with reliable activity against metallo-beta-lactamase inhibitors; third new CRAB drug, second new CRPA drug
2023	Durlobactam/sulbactam	No	Fourth new CRAB drug
2024	Cefepime/entmetazobactam	No	Eighth new CRE drug, third new CRPA drug
2024	Ceftobiprole	No	Fourteenth MRSA agent, multiple cephalosporins on the market
2024	Pivmecillinam	No	Available in Europe for decades; a drug for cystitis
2024	Sulopenem eztadroxil probenecid	No	Oral and covers ESBL, but only approved for bladder infections because was inferior in efficacy in complicated urinary tract and abdominal infection studies

Dates of new approvals are confirmed from the FDA Orange Book database (https://www.accessdata.fda.gov/scripts/cder/ob/index.cfm).

Abbreviations: CRAB, carbapenem-resistant *Acinetobacter baumannii*; CRE, carbapenem-resistant Enterobacterales; CRPA, carbapenem-resistant *Pseudomonas aeruginosa*; ESBL, extended-spectrum beta-lactamase–producing bacteria; FDA, Food and Drug Administration; MRSA, methicillin-resistant *Staphylococcus aureus*; NTM, nontuberculous mycobacteria; TB, tuberculosis; VRE, vancomycin-resistant *Enterococcus*.

Commercial failures of select antimicrobial developers (eg, Achaogen, which developed plazomicin; Nabriva, which developed lefamulin) have also been described as evidence of an antibiotic market failure. However, these developers charged premium pricing for antibiotics that did not address an unmet need (Table 2). Their failure is the result of a normally functioning for-profit market, not a market failure. The failure of plazomicin also highlights the risk that, in a normally functioning for-profit market, less desirable technology that initially addresses unmet need (eg, aminoglycoside that treats carbapenem-resistant Enterobacterales [CRE] but with substantial toxicity liability) may be superseded by subsequent technology with more desirable characteristics (eg, treat CRE with superior toxicity liability).

Thus, data do not support the assertion that we are in the midst of an antibiotic pipeline collapse. Rather, the world is considerably better positioned today regarding new antibiotics than we were 15 years ago. As such, current legislation seeking to appropriate $6 billion more in economic subsidies may be ill-timed.

### Are AMR-associated death rates driven by lack of antibiotics?

The astronomical number of AMR-associated deaths is frightening. However, a closer look at the methodology behind such numbers may moderate the conclusion. For example, the largest increase in deaths attributed to AMR were due to MRSA.^1^ Yet, since the year 2000, 14 new antibiotics that can treat MRSA infections have become clinically available (Table 2). Thus, these deaths are not driven by resistance to effective antibiotics, but rather reflect the mortality rate of serious infectious diseases despite existing effective treatments.

Similarly, other reported drivers of AMR-associated deaths were CRE, *Acinetobacter*, and *Pseudomonas*.^1^ However, since 2000, 8, 5, and 3 new antibiotics have become available that are effective against these pathogens, respectively (Table 2).

One could argue that, since these new antibiotics are so expensive, and outright unavailable in many countries, resistance still contributes to death. That fair argument belies the proposed solution. Rather than pouring more money into the pipeline to develop more antibiotics that will also be too expensive to be widely available, different solutions are required to enable widespread access to new antibiotics.

Attributing the magnitude of AMR-associated deaths to a dearth of new antibiotics makes the problem seem insurmountable and belittles successes achieved with the existing pipeline and incentives. Moreover, this narrative obfuscates the actual underlying conditions leading to many of these deaths: the pathophysiology and immunopathogenesis of the infections,^19^ poorly functioning health care systems, poor infection-control and antibiotic-use practices, and lack of access to new antibiotics in many geographical locations. These underlying conditions need to be acknowledged as we consider the best investment opportunities to address AMR infections.

## Sustainable solutions not requiring perpetual expansion of government appropriations

We agree with One Health recommendations to eliminate growth-promoting antibiotics in livestock; reduce antibiotic contamination in the environment; and curb antibiotic use without a prescription, which occurs in some low- and middle-income countries (LMICs).^3,15,20-22^ Indeed, prohibiting antibiotic dispensing without a prescription led to significant declines in antibiotic use in Saudi Arabia and Kerala, India.^20,21^

However, other practical, sustainable solutions to reduce inappropriate antibiotic use in humans have not been implemented (Table 3).^5,12-17,22^ Unfortunately these solutions have not been advocated for as vociferously as new appropriations to subsidize antibiotic development.

**Table 3.** Neglected, sustainable solutions to the AMR problem.

Solution	Process
Reduce antibiotic overuse	
Mandate public reporting of antibiotic use and link to pay for performance incentive for lower use and disincentive for higher use	Regulation or legislation
Focus research funds to reduce reliance on antibiotics: Vaccines to prevent bacteria/fungal infections, and AMR pathogens in particular Immunotherapies to prevent or treat bacterial/fungal infections Phages Microbiome-based strategies to prevent or eliminate AMR colonization Better infection-prevention implementation Short-course antibiotic therapy studies Rapid diagnostics and biomarkers to reduce antibiotic prescriptions Novel psychological approaches, such as nudges, to reduce antibiotic prescriptions	Regulation
Require special training or certification to prescribe new antibiotics and those addressing unmet need	Regulation or legislation
Ensuring future antibiotic R&D	
Establish a board including clinical experts to better target existing incentives	Legislation
Establish endowed nonprofits to conduct early stage discovery and R&D of AMR solutions	Legislation

Abbreviations: AMR, antimicrobial resistance; R&D, research and development.

### Strategies to Decrease Antibiotic Usage

First, the success of pay-for-performance measures at reducing health care–associated infection rates in the United States demonstrates the power of aligning market forces with desired outcomes.^5,16,17,23^ We should mandate publicly reporting rates of antibiotic usage in acute and ambulatory care settings on a per-patient basis. Incentives can be implemented for high-performing health systems (lower rates of antibiotic used per patient per day), the costs of which are offset by disincentives for lower performing systems. Such a mechanism aligns the financial interests of health systems with the public interest in reducing inappropriate antibiotic use.

One potential concern about publicly reporting antibiotic prescriptions is enabling patients to choose practitioners who are more likely to prescribe antibiotics. However, baseline prescribing rates are so high generally that such an effect is unlikely to worsen prescriptions overall. Furthermore, the financial penalties from overprescribing would have a natural tendency to ameliorate this risk.

Second, focus existing research funding on the development of novel strategies that prevent and treat infections, reducing the need to use antibiotics. Examples include the following: molecular diagnostics, vaccines, immunotherapies, and phages targeting AMR pathogens; microbiome-based strategies to ameliorate AMR colonization; more effective implementation of infection-prevention methods including in LMICs; shorter durations of therapy; and psychological strategies to reduce antibiotic prescriptions.^24-26^

Third, enact new regulation and/or law that requires completion of explicit training in antibiotic stewardship before licensed practitioners (physicians, allied health professionals, dentists, or veterinarians) are allowed to prescribe newly approved antibiotics that address unmet AMR needs.^16,17,23,27^ Antibiotics are the most powerful life-saving interventions in medicine.^11,14,28^ Unfortunately, the more they are used, the less effective they become. It is a tremendous failure of public policy that we allow anyone with a license to prescribe any antibiotic, at any time, for any duration, thereby accelerating the erosion of their life-saving power.

Skeptics may doubt that requiring training in antibiotic stewardship will improve prescribing habits. However, clinicians have been inappropriately prescribing antibiotics since penicillin first became available in the early 1940s.^29^ Eighty years later, modern studies continue to demonstrate rampant misuse of antibiotics, with approximately half of antibiotics prescribed in the United States being unnecessary; for some diseases, upwards of 80% are unnecessary.^30,31^ Furthermore, studies have demonstrated repeatedly that implementation of antibiotic stewardship programs reduces unnecessary antibiotic prescriptions without causing harm from undertreatment.^32-36^ While training may not result in perfect prescribing habits, ample evidence indicates that it improves appropriate antibiotic prescribing.

Governments already have regulatory authority to restrict prescriptions of specific drugs based on safety concerns (eg, oncology products, risk evaluation and mitigation strategies, etc). However, whether existing regulations can be used to enforce antibiotic-prescribing restrictions based on AMR public health concerns remains unclear. New legislation may be required.

Requiring specialized training and certification to prescribe critical antibiotics necessitates having adequate workforce available. Unfortunately, there is a lack of experts trained in antibiotic stewardship and infectious diseases,^37^ and nearly half of all US infectious diseases training programs went unfilled in 2023, continuing a worsening year-over-year trend.^38^ The reason is simple: infectious diseases specialists earn an average of $20 000 less per year than general internists, who did not complete 2 extra years of training to subspecialize.^39^ Requiring specialized training in antimicrobial stewardship to prescribe needed, new antibiotics will help address this workforce shortage by creating a market force in which health systems seek to hire or contract with such experts.^23^ As well, expansion of telemedicine services would enable experts in antimicrobial prescribing to serve as consultants to clinicians in large geographical areas.^40^

Any concerns about requiring specialized training in antibiotic stewardship to prescribe new antibiotics must be balanced against the public good of diminishing antibiotic resistance.^22,34,41^ Antibiotics are a shared societal trust: every individual's use affects the ability of everyone else in society to have them available to cure their diseases.^27^ We must act responsibly to preserve their life-saving power for as long as possible.

### Strategies to Ensure Future R&D of Antibiotics

We believe that it is problematic to call for additional taxpayer-funded economic subsidies to support antibiotic development by for-profit companies that then price the new antibiotics so highly that it makes them unavailable. In such cases, taxpayers subsidize the costs of R&D on the front end only to pay premium pricing on the back end to access the drugs. We do not believe that creating a completely artificial market sustained entirely by taxpayer subsidies is a responsible, or sustainable, solution to AMR.

We specifically disagree with the concept of a “subscription model” of subsidy as described in the PASTEUR Act. In such a model, after a new antibiotic is approved for use, companies are paid a flat rate by governments to access it, whether the antibiotic is used or not, and whether or not taxpayer subsidies were used to develop the antibiotic. This model has been colloquially referred to as a “Netflix” model. Such a subscription model has a favorable economic value for taxpayers when we want to encourage as much use of a drug as possible, such as for therapies for HIV or hepatitis C, to limit transmission and spread to others. In contrast, the interest of the public is to limit antibiotic use to preserve their effectiveness for as long as possible. A model in which a flat rate is paid to a company to purchase the right to use as little of the drug as possible is a wonderful financial arrangement for the company selling the drug, but a terrible value for the customer in this relationship—ie, the public/taxpayers. If one watched as little programming as possible, one would not keep paying the monthly Netflix subscription fee. We have instead sardonically referred to this model as a gym membership model, where paying monthly fees makes people feel psychologically like they are doing something to benefit their health even if they don't often go to the gym.

Another analogy that has been used regarding such an antibiotic subsidy arrangement is that of a fire extinguisher. People purchase fire extinguishers in advance so that, if a fire does break out, the fire extinguisher is already available. This analogy is gratifying for antibiotics on a superficial level; we should pay for antibiotics even if we plan to rarely use them, so that when we do need to use them, they are available. However, under a modicum of scrutiny, this analogy breaks down. Industry is charging $10 000 or more for a standard treatment course of new antibiotics.^42^ In contrast, fire extinguishers are commodities that cost $20 to $30 on Amazon, and are reusable multiple times for that low cost. Instead of paying per use, the PASTEUR Act would appropriate $6 billion to subsidize purchase of such drugs in a subscription model, despite the fact that the average annual sales of new antibiotics is a meager 5% of that number.^12^ No one would propose a $6 billion economic subsidy for the fire extinguisher industry.

The PASTEUR Act has been introduced into 3 consecutive US Congresses and still not passed. Thus, policymakers appear to share our concerns about the value of such an arrangement. Even if the bill's advocates disagree with us about its importance, there is a practical consideration regarding the futility of spending more effort on it. Such effort is better spent focusing on alternative solutions that are more palatable to policymakers, saving efforts to push for additional economic subsidies for a time when the pipeline actually is collapsing, as it did 15 years ago. Indeed, history suggests that a third pipeline collapse may occur at some point in the future, because we have once again brought to market many antibiotics that have similar/overlapping spectra of activity (Table 2).

For now, rather than creating new subsidies, we propose to better target existing incentives.^13^ For example, one of the most pressing current unmet needs is for a new orally administered antibiotic that can treat resistant gram-negative bacteria, yet existing incentives have failed to produce such an agent, and instead have subsidized development of redundant parenteral agents. The PASTEUR Act would create an advisory board to maintain a list of AMR targets for the proposed new incentive. We agree that such a board should be established, because it is needed to help national governments better target existing economic incentives (eg, towards oral antibiotics specifically).^13^ Clinicians who practice in diverse settings should staff this advisory board.

As an alternative to an entirely artificial market perpetually sustained by taxpayer subsidies, we previously described an endowed nonprofit model that could undertake early-phase R&D and establish a bench of molecules in waiting.^12^ For one-sixth of the costs appropriated in the PASTEUR Act, 2 or 3 nonprofit companies could be endowed to compete in sustainably conducting antibiotic discovery and early-phase development, without requiring continuous subsidies over time.^12^ This amount of funding would enable adequate and sustainably renewing annual operating costs from interest earned off the base endowment. As resistance evolves, molecules that target unmet needs could be pulled from the bench to partner with for-profit companies to complete pivotal clinical trials and facilitate market entry. This strategy de-risks development for industry and minimizes the tremendous cost of economic discounting, a primary demotivating factor in for-profit drug development.^12,43^ The endowed nonprofit companies are intended to bolster and stabilize the for-profit drug market rather than replace it.

Existing nonprofits, such as The Global Alliance for TB Drug Development and the Global Antibiotic Research and Development Partnership (GARDP), have demonstrated their capability of driving new antibiotic development. However, these organizations have focused on funding and conducting clinical trials of previously discovered molecules, rather than conducting initial discovery and preclinical development.

## Conclusion

Antimicrobial resistance represents a sustained, continuous threat, although temporarily less acute today than in 2009 due to solutions advocated for years ago. There is a natural tendency to continuously amplify threats, to compete for attention and resources among the chorus of voices. However, hyperbole can erode credibility among policymakers and taxpayers, who bear the burden of the cost of proposed solutions.

Microbes maintained antimicrobial efficacy for 2 billion years before humans got involved. They did so by judiciously stewarding antibiotic use. If microbes are smart enough to do it, we hope humans can be too. Rather than a war, we must seek to achieve peaceful coexistence with normal microbial flora by using as few antibiotics as are necessary to restore health.

We support rational regulatory and legislative changes to discourage inappropriate antibiotic use in humans and agriculture. We also support better targeting existing economic incentives, and we believe a one-time taxpayer investment to establish several sufficiently endowed nonprofit companies to self-fund R&D over time is a more rational and achievable means of sustaining antibiotic R&D in the long run.

## Supplementary Material

qxaf012_Supplementary_Data
